# Supplementary material for: Role and mechanism of NCAPD3 in promoting malignant behaviors in gastric cancer
Source: Front Pharmacol. 2024 Apr 22;15:1341039. doi: 10.3389/fphar.2024.1341039 (PMC11070777; doi:10.3389/fphar.2024.1341039)
Supplement: Supplementary file 11 [file DataSheet2.ZIP › GSEA/GO_CC/my_analysis.Gsea.1599461408869/GO_APICAL_PLASMA_MEMBRANE.html]

Details for gene set GO\_APICAL\_PLASMA\_MEMBRANE[GSEA]

|  || Dataset | filtered\_dataset.sample\_info.cls#WT\_versus\_NCAPD3\_MUT |
| Phenotype | sample\_info.cls#WT\_versus\_NCAPD3\_MUT |
| Upregulated in class | WT |
| GeneSet | GO\_APICAL\_PLASMA\_MEMBRANE |
| Enrichment Score (ES) | 0.24773864 |
| Normalized Enrichment Score (NES) | 1.1451987 |
| Nominal p-value | 0.28140703 |
| FDR q-value | 0.88580596 |
| FWER p-Value | 1.0 |
Table: GSEA Results Summary

  

Fig 1: Enrichment plot: GO\_APICAL\_PLASMA\_MEMBRANE      
 Profile of the Running ES Score & Positions of GeneSet Members on the Rank Ordered List

  

| SYMBOL | TITLE | RANK IN GENE LIST | RANK METRIC SCORE | RUNNING ES | CORE ENRICHMENT || 1 | 659 | BMPR2 | 16 | 1.047 | 0.0605 | Yes |
| 2 | 10207 | INADL | 73 | 0.851 | 0.0786 | Yes |
| 3 | 7976 | FZD3 | 88 | 0.815 | 0.1246 | Yes |
| 4 | 92140 | MTDH | 93 | 0.801 | 0.1768 | Yes |
| 5 | 56288 | PARD3 | 99 | 0.790 | 0.2276 | Yes |
| 6 | 6573 | SLC19A1 | 231 | 0.650 | 0.1777 | Yes |
| 7 | 5728 | PTEN | 285 | 0.606 | 0.1811 | Yes |
| 8 | 538 | ATP7A | 305 | 0.588 | 0.2079 | Yes |
| 9 | 960 | CD44 | 339 | 0.573 | 0.2234 | Yes |
| 10 | 1080 | CFTR | 366 | 0.557 | 0.2430 | Yes |
| 11 | 259266 | ASPM | 411 | 0.531 | 0.2477 | Yes |
| 12 | 23255 | MTCL1 | 505 | 0.471 | 0.2130 | No |
| 13 | 153562 | MARVELD2 | 732 | 0.359 | 0.0745 | No |
| 14 | 57619 | SHROOM3 | 783 | 0.331 | 0.0612 | No |
| 15 | 6558 | SLC12A2 | 839 | 0.276 | 0.0405 | No |
| 16 | 6520 | SLC3A2 | 878 | -0.290 | 0.0330 | No |
| 17 | 8140 | SLC7A5 | 890 | -0.305 | 0.0461 | No |
| 18 | 4008 | LMO7 | 909 | -0.335 | 0.0561 | No |
| 19 | 301 | ANXA1 | 914 | -0.342 | 0.0768 | No |
| 20 | 4680 | CEACAM6 | 955 | -0.374 | 0.0736 | No |
| 21 | 9180 | OSMR | 981 | -0.394 | 0.0827 | No |
| 22 | 9076 | CLDN1 | 1084 | -0.463 | 0.0409 | No |
| 23 | 9962 | SLC23A2 | 1169 | -0.537 | 0.0172 | No |
| 24 | 1956 | EGFR | 1266 | -0.657 | -0.0069 | No |
| 25 | 57835 | SLC4A5 | 1319 | -0.736 | 0.0062 | No |
| 26 | 5794 | PTPRH | 1368 | -0.854 | 0.0303 | No |
Table: GSEA details [plain text format]

  

Fig 2: GO\_APICAL\_PLASMA\_MEMBRANE      
 Blue-Pink O' Gram in the Space of the Analyzed GeneSet

  

Fig 3: GO\_APICAL\_PLASMA\_MEMBRANE: Random ES distribution      
 Gene set null distribution of ES for **GO\_APICAL\_PLASMA\_MEMBRANE**
